# Efficacy and safety during extended treatment of lesinurad in combination with febuxostat in patients with tophaceous gout: CRYSTAL extension study

**DOI:** 10.1186/s13075-018-1788-4

**Published:** 2019-01-07

**Authors:** Nicola Dalbeth, Graeme Jones, Robert Terkeltaub, Dinesh Khanna, Maple Fung, Scott Baumgartner, Fernando Perez-Ruiz

**Affiliations:** 1 Department of Medicine, University of Auckland, 85 Park Road, Grafton, Auckland, 1 New Zealand; 2 University of Tasmania, Hobart, TAS Australia; 3 VA Healthcare System, University of California, San Diego, CA USA; 4 University of Michigan, Ann Arbor, MI USA; 5 Present address: Arena Pharmaceuticals, Inc, San Diego, CA USA; 6 Former address: Ardea Biosciences, Inc, San Diego, CA USA; 7 Present address: drB Consulting, LLC, Spokane, WA USA; 8 Hospital Universitario Cruces, Baracaldo, Vizcaya Spain

**Keywords:** Extension study, Febuxostat, Gout, Lesinurad, Phase III trial, Serum urate, Tophus

## Abstract

**Background:**

In gout, long-term urate-lowering therapy (ULT) promotes dissolution of tissue urate crystal deposits. However, no studies using combined xanthine oxidase inhibition and uricosuric ULT have focused on clinical outcomes or adverse events (AEs) beyond 12 months of therapy. Our objective in the present study was to examine efficacy and long-term safety in patients with tophaceous gout receiving febuxostat plus lesinurad as combination therapy.

**Methods:**

Patients receiving combined lesinurad and febuxostat in the 12-month core CRYSTAL study continued at the same doses in the extension study (“200CONT”, “400CONT”), whereas those receiving only febuxostat 80 mg were randomized to lesinurad 200 or 400 mg with febuxostat (“200CROSS”, “400CROSS”). The primary endpoint was the proportion of patients experiencing complete resolution (CR) of at least one target tophus by extension month (EM) 12. The key secondary endpoint was mean rate of gout flares requiring treatment from the end of EM 2 to the end of EM 12. Secondary endpoints included reduction in the sum of areas for all target tophi. Safety assessments included AEs and laboratory data for the entire extension study (median length of lesinurad exposure, 800 days).

**Results:**

Of 235 patients completing the core study, 196 (83.4%) enrolled in the extension: 200CONT (*n* = 64), 200CROSS (*n* = 33), 400CONT (*n* = 65), and 400CROSS (*n* = 34). At EM 12, 59.6%, 43.5%, 66.7%, and 50.0% of patients, respectively, had CR of at least one target tophus. The sum of areas for all target tophi was reduced by 76.4%, 58.1%, 77.5%, and 62.8%, respectively. The adjusted mean (SE) rates of gout flares requiring treatment from the end of EM 2 to the end of EM 12 were 0.6 (0.19), 1.3 (0.48), 0.2 (0.08), and 1.9 (0.93), respectively. Target sUA < 5.0 mg/dl was achieved by 77.1%, 79.2%, 88.5%, and 71.4% of patients, respectively. Exposure-adjusted incidence rates of treatment-emergent adverse events (TEAEs) and renal-related TEAEs in the core study were not increased with prolonged lesinurad exposure in the extension study.

**Conclusions:**

Patients receiving lesinurad plus febuxostat therapy for 2 years continued to be at sUA target. Patients exhibited a progressive increase in CR of at least one target tophus, progressive reduction in tophus size, and reduction of gout flares requiring treatment over the second year, with AEs consistent with those observed in the core study.

**Trial registration:**

ClinicalTrials.gov, NCT01510769. Registered on 13 January 2012.

**Electronic supplementary material:**

The online version of this article (10.1186/s13075-018-1788-4) contains supplementary material, which is available to authorized users.

## Background

Gout is characterized by increased body urate stores, reflected in elevated serum urate (sUA) levels (hyperuricemia), and deposition of monosodium urate crystals in joints and other tissues. Gout causes recurrent flares of acute inflammatory arthritis. When hyperuricemia is inadequately treated in gout, the disease can progress over time to palpable tophus formation and chronic erosive arthritis [1]. The goal of long-term management of gout is to reduce and maintain sUA below saturating levels (> 6.8 mg/dl), typically < 6.0 mg/dl, or < 5.0 mg/dl for patients with more severe gout, including palpable tophi [1, 2]. Sustained lowering of sUA reduces the volume of monosodium urate crystals, lowers the incidence of gout flares, and promotes tophus resolution [3–5]. The recommended first-line urate-lowering therapy (ULT) is a xanthine oxidase inhibitor, either allopurinol or febuxostat [1, 2], in order to lower production of urate [6]. For patients who cannot achieve their sUA target with a medically appropriate dose of a xanthine oxidase inhibitor, treatment guidelines provide the option of combining the xanthine oxidase inhibitor with a uricosuric agent [1, 2]. Uricosuric therapies target renal underexcretion of uric acid, which contributes to hyperuricemia in most people with gout [7].

Lesinurad is a uricosuric agent recently approved in the United States and Europe at a 200-mg oral dose in combination with a xanthine oxidase inhibitor for the treatment of hyperuricemia associated with gout in patients who fail to achieve sUA target with a xanthine oxidase inhibitor alone [8]. Lesinurad inhibits the urate transporter URAT1, which is responsible for reabsorption of urate from the renal tubule lumen [9]. By inhibiting URAT1, le-sin-ur-ad increases excretion of uric acid and thereby lowers sUA [10, 11]. Lesinurad thereby offers a complementary mechanism of action to xanthine oxidase inhibitors. Although lesinurad alters the clearance of oxypurinol, the active metabolite of allopurinol [12], lesinurad does not alter the clearance of febuxostat [10].

The efficacy and safety of lesinurad (200 or 400 mg, orally, once daily) in combination with febuxostat 80 mg compared with febuxostat 80 mg alone was investigated in patients with gout and palpable tophi (“tophaceous gout”) in a 12-month, randomized, double-blind, placebo-controlled, multicenter study (CRYSTAL [Combination Treatment Study in Subjects with Tophaceous Gout with Lesinurad and Febuxostat]; ClinicalTrials.gov, NCT01510769). The primary study endpoint was the proportion of patients achieving the sUA target of < 5.0 mg/dl at the month 6 visit. Lesinurad 400 mg in combination with febuxostat 80 mg significantly increased the proportion of patients achieving the sUA target at the month 6 visit versus febuxostat 80 mg alone (*p* < 0.0001), whereas lesinurad 200 mg daily plus febuxostat 80 mg did not (*p* = 0.13) [13]. At all other time points, more patients in the lesinurad 200-mg group achieved the sUA target. The safety profile of lesinurad at the approved 200-mg dose combined with febuxostat 80 mg was comparable to febuxostat 80 mg alone, except for the higher incidence of predominantly reversible serum creatinine (sCr) elevations. The present study assessed the efficacy and long-term safety of lesinurad in combination with febuxostat in patients who completed CRYSTAL and entered an extension study (NCT01808144).

## Methods

### Study design and medications

The CRYSTAL extension study was performed at 73 study sites in six countries: United States, Canada, Poland, Switzerland, Australia, and New Zealand. The study was conducted in accordance with the ethical principles of good clinical practice according to the International Conference on Harmonisation Harmonised Tripartite Guideline. Patients provided informed written consent and had the right to withdraw at any time.

**Fig. 1:**
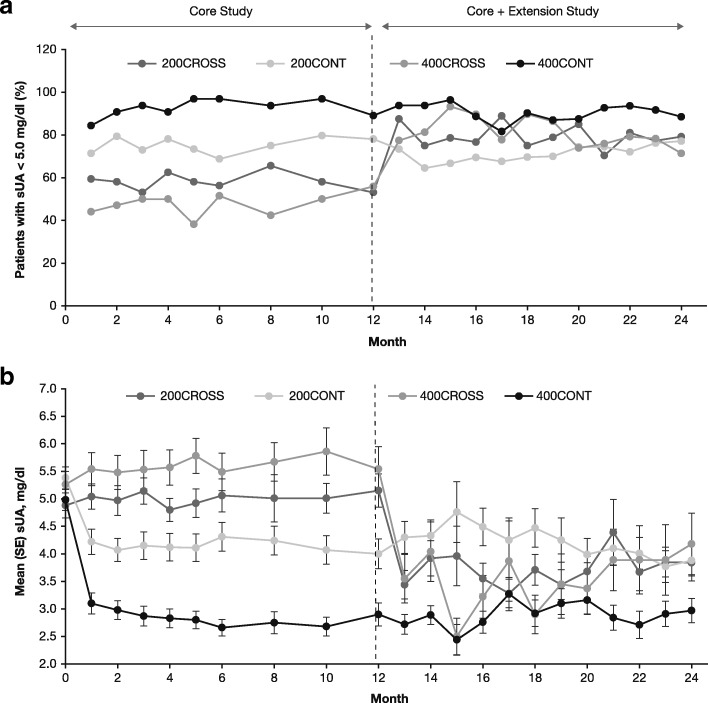
Proportion of patients with serum urate (sUA) < 5.0 mg/dl (**a**) and mean (SE) sUA levels (**b**) in the core study and extension study: observed cases (intention-to-treat population). *CONT* Continuation of lesinurad treatment, *CROSS* Crossover from core study placebo to lesinurad treatment

All patients who completed treatment in the 12-month CRYSTAL study (“core study”) were eligible to enter the optional extension study, which was planned to include a treatment period of up to approximately 5 years. Patients who completed the core study [13] on lesinurad 200 mg plus febuxostat 80 mg or lesinurad 400 mg plus febuxostat 80 mg continued at the same lesinurad and febuxostat doses in the extension study (“200CONT” and “400CONT,” respectively), without scheduled interruption in study medications (Fig. 1). Patients who completed the core studies on febuxostat 80 mg alone (placebo) were randomized 1:1 using an interactive voice/web response system to receive either lesinurad 200 mg plus febuxostat 80 mg or lesinurad 400 mg plus febuxostat 80 mg in the extension study (“200CROSS” and “400CROSS,” respectively). The lesinurad dose was blinded until database lock of the core study and all patients had reached at least month 12 in the extension study, after which patients transitioned to open-label treatment. No efficacy measurements were planned beyond month 12 of the extension, whereas safety was followed throughout the extension. In a protocol amendment to the extension study, following a review of the efficacy and safety data at interim (12-month) analysis, patients receiving lesinurad 400 mg were decreased to the 200-mg daily dose; the efficacy analyses were completed before the dose change.

Gout flare prophylaxis was given to all patients in the extension study to maintain blinding of the core study. As in the core study, flare prophylaxis consisted of colchicine 0.5 or 0.6 mg daily (depending on local dose per tablet) or a nonsteroidal anti-inflammatory drug with or without a proton pump inhibitor if patients had an intolerance/contraindication to colchicine. Prophylaxis was administered from the extension baseline visit (day 1) through extension month 2; continuation after extension month 2 was at the discretion of investigators but was not to exceed 6 months. In cases of sCr more than 1.5 times baseline, patients were advised to drink ≥ 2000 ml of fluids, and if urine pH was < 6.5, investigators were to consider initiating a urinary alkalinizing agent. Treatment compliance was assessed by maintaining medication-dispensing records and by verification of returned and remaining study medications.

### Patients

For entry to the 12-month core CRYSTAL study, patients with gout aged 18–85 years on ULT currently or in the past, as well as those who had never received a ULT, were eligible. sUA was required to be ≥ 8.0 mg/dl for patients not receiving ULT and ≥ 6.0 mg/dl for those receiving ULT. Patients were also required to have at least one measurable tophus on the hands/wrists and/or feet/ankles ≥ 5 and ≤ 20 mm in the longest diameter (length). Patients were given febuxostat 80 mg daily for 3 weeks before randomization to lesinurad (200 or 400 mg daily) or placebo added to febuxostat. To enter the extension study, patients must have completed treatment in the core study.

Patients were discontinued from the extension study if they withdrew consent, developed an adverse event (AE) or laboratory abnormality requiring treatment discontinuation, experienced an sCr elevation three or more times baseline before the first lesinurad dose or ≥ 4 mg/dl or an estimated creatinine clearance < 30 ml/min on repeated measurements, or developed a kidney stone.

### Assessments

Baseline assessments for the extension study were performed at the month 12 visit of the core study. Assessments for efficacy and safety during the extension study were performed monthly at months 1–12 (blinded phase), and safety assessments were additionally performed every subsequent 2 months until patient withdrawal or study termination (open-label phase). A termination visit occurred within ~ 14 days after the last dose of lesinurad.

### Efficacy assessments

Efficacy assessments in the core studies were continued through month 12 of the extension study. The primary endpoint of the extension study was the proportion of patients who experienced complete resolution of at least one target tophus at each monthly visit and overall. Secondary tophus endpoints were the proportion of patients with best tophus response (complete or partial resolution) of at least one target tophus at each visit and overall, and the mean change and mean percentage change from core study baseline in the sum of areas for all target tophi at each visit.

The key secondary endpoint was the mean rate of gout flares requiring treatment in the period from the end of extension month 2 to the end of extension month 12, recorded by patients via electronic diary. Secondary endpoints included the proportion of patients with sUA < 5.0 mg/dl (primary endpoint in the core study), as well as < 4.0 and < 3.0 mg/dl, at each monthly visit up to month 12 in the extension study. Absolute and percentage changes in sUA from the core study baseline were also measured at each visit.

### Safety assessments

Safety assessments throughout the extension study included the incidence and severity of treatment-emergent adverse events (TEAEs; coded according to Medical Dictionary for Regulatory Activities version 14.0) and their relationship to study medication, TEAEs leading to withdrawal, and serious adverse events (SAEs). Renal safety assessments consisted of renal-related and kidney stone TEAEs (itemized in Additional file 1: Table S1) and instances of sCr elevation greater than or equal to 1.5 times and greater than or equal to 2.0 times baseline. Assessments of cardiovascular TEAEs included cardiovascular-related AEs and rates of major adverse cardiovascular events (MACE) (i.e., cardiovascular death, nonfatal myocardial infarction, and nonfatal stroke) [14]. Clinical laboratory evaluations (hematology, serum chemistry, and urinalysis) and vital sign assessments were performed at planned monthly assessments. Safety data were reviewed by an independent data monitoring committee, renal events adjudication committee, and cardiovascular endpoints adjudication committee appointed by the study sponsor.

### Statistical analysis

Efficacy and safety analyses used the intention-to-treat and safety populations, respectively. The intention-to-treat population included all randomized patients who received at least one dose of lesinurad in the extension study and had at least one postbaseline efficacy measurement. The safety population included all patients who received at least one dose of lesinurad in the extension study.

Tophus resolution rates were compared between treatment groups in each time interval and tested using the Cochran–Mantel–Haenszel test statistic, stratifying by core study to account for any study-specific sUA-lowering effect and by core day − 7 renal function (randomized values). Rates of gout flares requiring treatment were compared between groups by adding the number of flares in each time interval. Estimates of the adjusted rate of gout flares requiring treatment were obtained from negative binomial regression adjusted by day − 7 renal function and day − 7 sUA status (randomized values) and log follow-up time as the offset variable.

Proportions of patients at each scheduled visit who achieved sUA < 5.0, < 4.0, and < 3.0 mg/dl were tabulated for each treatment group. Analyses used missing value imputation (i.e., patients with missing data were considered nonresponders), observed cases, and last observation carried forward imputation. sUA levels during the extension study were analyzed using an analysis of covariance model with baseline value as a covariate and treatment group and core day − 7 sUA status as factors (randomized values) for the intention-to-treat population.

TEAEs were defined as AEs that started on or after the first lesinurad dose in the extension study or AEs that started before the first lesinurad dose but worsened during the extension study. TEAEs are presented as cumulative results throughout the extension study. TEAEs were also calculated as exposure-adjusted incidence rates (number of patients with events per 100 person-years [PY]) to adjust for differences in duration of lesinurad exposure in the four treatment groups. Exposure-adjusted incidence rates are presented for the core study and for the core plus 12 months of the extension study.

Descriptive statistics are provided for safety data, with categorization according to randomized treatment group. For patients whose lesinurad dose was decreased from 400 to 200 mg daily at extension month 12, according to protocol amendment, safety analyses are allocated to the initial randomized dose. All analyses were performed with SAS software version 9.1.3 or higher (SAS Institute, Cary, NC, USA).

## Results

### Patient disposition

Of the 324 patients who enrolled in the core study, 235 (72.5%) completed the 12 months of treatment. A total of 196 patients (83.4%) enrolled in the extension study and received at least one dose of lesinurad (Additional file 1: Figure S1). There were 149 (76.0%) patients who completed the study through extension month 12 and 124 (63.3%) who completed through extension month 24. Altogether, 88 patients (44.9%) discontinued the study early, with similar percentages in all treatment groups. The most common reasons for early study termination were consent withdrawal (18.8% of patients in 200CONT, 12.1% in 200CROSS, 26.2% in 400CONT, and 17.6% in 400CROSS, respectively) and AEs (14.1% of patients in 200CONT, 6.1% in 200CROSS, 13.8% in 400CONT, and 17.6% in 400CROSS). Demographic and baseline characteristics at the time of core study initiation were similar across the extension treatment groups (Table 1).

**Table 1:** Demographic and baseline characteristics of extension study groups at the start of the core study (safety population)

Parameter	200CONT (*n* = 64)	200CROSS (*n* = 33)	400CONT (*n* = 65)	400CROSS (*n* = 34)
Age, years, mean (SD)	53.2 (10.4)	52.4 (10.1)	52.1 (11.5)	52.1 (10.6)
Male sex, *n* (%)	63 (98.4)	33 (100)	60 (92.3)	32 (94.1)
Race, *n* (%)				
Asian	5 (7.8)	3 (9.1)	5 (7.7)	1 (2.9)
Black or African American	9 (14.1)	4 (12.1)	8 (12.3)	1 (2.9)
Māori	0	0	2 (3.1)	0
White	48 (75.0)	26 (78.8)	49 (75.4)	31 (91.2)
Body mass index, kg/m^2^, mean (SD)	31.9 (5.9)	33.9 (6.1)	31.8 (5.8)	33.4 (5.9)
Duration since gout diagnosis, years, mean (SD)	15.8 (9.5)	13.8 (9.0)	12.9 (10.0)	12.0 (9.3)
Number of gout flares in past 12 months, mean (SD)	7.7 (13.6)	7.1 (5.8)	8.1 (8.2)	7.1 (5.9)
Type of gout flare prophylaxis, *n* (%)				
Colchicine	55 (85.9)	24 (72.7)	53 (81.5)	30 (88.2)
NSAID	6 (9.4)	3 (9.1)	12 (18.5)	4 (11.8)
Both	1 (1.6)	0	2 (3.1)	0
Other/missing	4 (6.3)	6 (18.2)	2 (3.1)	0
sUA, mg/dl, mean (SD)				
At core study baseline^a^	5.4 (1.6)	4.9 (1.3)	5.0 (1.5)	5.3 (1.4)
At extension study start	4.0 (2.2)	5.2 (1.7)	2.9 (1.7)	5.5 (2.4)

*Abbreviations: CONT* Continuation of lesinurad treatment, *CROSS* Crossover from core study placebo to lesinurad treatment, *NSAID* Nonsteroidal anti-inflammatory drug, *sUA* Serum urate

^a^Following 3 weeks of febuxostat 80 mg daily

### Study medications

The median [range] of lesinurad exposure in days (inclusive of dose interruptions) in the extension study was comparable between the four groups (200CONT, 792 [39, 1154]; 200CROSS, 799 [1, 1178]; 400CONT, 826 [36, 1191]; 400CROSS, 776 [12, 1084]). Lesinurad exposure in the core plus extension was greater in the CONT groups (200CONT, 1134 [373, 1495]; 400CONT, 1155 [372, 1523]) than the CROSS groups (200CROSS, 799 [1, 1178]; 400CROSS, 776 [12, 1084]). Expressed as PY, lesinurad exposure in core plus extension studies was 173.6, 61.9, 180.6, and 61.7 PY in the 200CONT, 200CROSS, 400CONT, and 400CROSS groups, respectively. Median compliance with lesinurad during the extension study was > 97% in all groups. The lesinurad dose was decreased, per the protocol amendment, from 400 to 200 mg/day in 47 patients.

### Efficacy assessments

#### Tophus resolution and area reduction

The proportion of patients with complete resolution of at least one target tophus increased in all groups with exposure to combination therapy in the extension study (Fig. 1a). By month 12 in the extension study, 59.6%, 43.5%, 66.7%, and 50.0% of patients in the 200CONT, 200CROSS, 400CONT, and 400CROSS groups, respectively, had complete resolution of at least one target tophus. The proportions of patients who experienced complete or partial resolution of at least one target tophus by month 12 of the extension study were 74.5%, 82.6%, 84.3%, and 80.8%, respectively.

The percentage reductions from the core study baseline in the sum of the areas for all target tophi were 76.4%, 58.1%, 77.5%, and 62.8% for the 200CONT, 200CROSS, 400CONT, and 400CROSS groups, respectively, at extension month 12 (Fig. 1b). The difference in the percentage reduction between 200CROSS and 200CONT (− 21.55 [95% CI, – 45.44, 2.35]) was not significant (*p* = 0.076), whereas the difference between 400CROSS and 400CONT (− 15.98 [95% CI, – 42.72, 10.75] was not different (*p* = 0.24).

#### Gout flares requiring treatment

The adjusted mean (SE) rates of gout flares requiring treatment from the end of extension month 2 to the end of extension month 12 were 0.6 (0.19) for 200CONT, 1.3 (0.48) for 200CROSS, 0.2 (0.08) for 400CONT, and 1.9 (0.93) for 400CROSS. The adjusted rate was 90% lower with 400CONT than with 400CROSS (incidence rate ratio [95% CI] CROSS vs. CONT = 0.1 [0.0–0.4]; *p* = 0.0007) but was not significantly lower with 200CONT than with 200CROSS (incidence rate ratio = 0.5 [0.2–1.2]; *p* = 0.13).

**Fig. 2:**
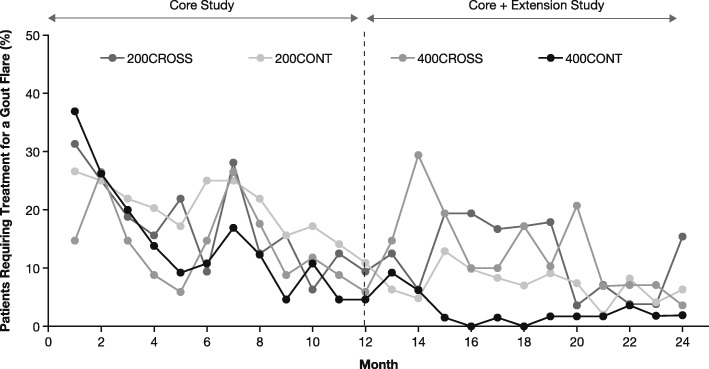
Percentage of patients with gout flare requiring treatment in intention-to-treat population receiving lesinurad + febuxostat up to 24 months. *CONT* Continuation of lesinurad treatment, *CROSS* Crossover from core study placebo to lesinurad treatment

The proportion of patients requiring treatment for a gout flare generally decreased over time in the core and extension studies for all groups (Fig. 2). Proportions of patients with a gout flare requiring treatment between extension months 2 and 12 were lowest in the 400CONT group (9.2%) and highest in the 400CROSS and 200CONT groups (35.5%), with the 200CROSS group being intermediate (27.4%).

#### sUA endpoints

**Fig. 3:**
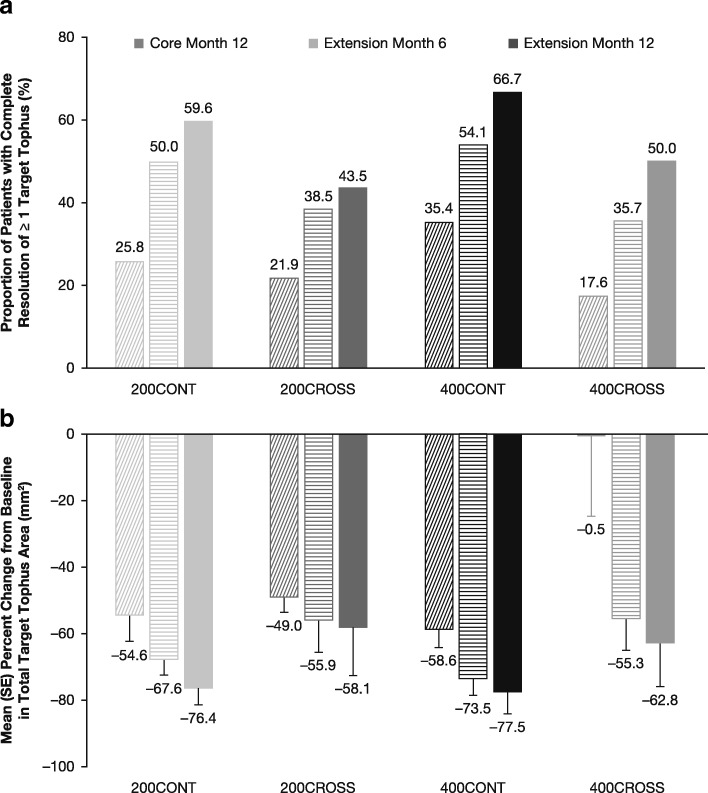
Proportion of patients with complete resolution of at least one target tophus (**a**) and percentage mean change from baseline in target tophus area (**b**) up to 24 months on lesinurad + febuxostat: observed cases. *CONT* Continuation of lesinurad treatment, *CROSS* Crossover from core study placebo to lesinurad treatment

The proportion of patients with sUA < 5.0 mg/dl during the core study ranged from 68.8% to 79.7% in the 200CONT group and 84.4% to 96.9% in the 400CONT group (Fig. 3a). These proportions were maintained during 12 months of the extension study in both the 200CONT (range, 64.5–77.1%) and 400CONT (range, 81.6–93.6%) groups. In the 200CROSS and 400CROSS groups, the proportion of patients with sUA < 5.0 mg/dl at each study visit ranged from 53.1% to 65.6% and from 38.2% to 55.9%, respectively, during the core study while receiving febuxostat alone. This proportion increased during the extension study in both the 200CROSS (range, 70.4–88.9%) and 400CROSS (range, 71.4–93.3%) groups. The same trends were also observed with targets of < 4.0 and < 3.0 mg/dl (data not shown).

At the end of the core studies, mean sUA was significantly lower in patients treated with combined lesinurad and febuxostat than in those treated with febuxostat alone (*p* < 0.0001, all group comparisons) (Fig. 3b). In the extension study, mean sUA levels in the crossover groups were reduced after 1 month of lesinurad treatment; in subsequent months, sUA levels were similar between the 200CONT and 200CROSS groups and between the 400CONT and 400CROSS groups. After 12 months in the extension study, mean (SD) sUA levels were 3.9 [1.9], 3.8 [1.6], 3.0 [1.6], and 4.2 [3.0] mg/dl for the 200CONT, 200CROSS, 400CONT, and 400CROSS groups, respectively.

### Safety assessments

#### Adverse events

At least one TEAE was experienced in the extension study by 78.1%, 81.8%, 87.7%, and 97.1% of the 200CONT, 200CROSS, 400CONT, and 400CROSS groups, respectively. Serious TEAEs were reported in 14.1%, 9.1%, 13.8%, and 14.7% of the respective groups, and TEAEs led to study withdrawal in 15.6%, 6.1%, 14.8%, and 14.7%. The most common individual TEAEs (in > 5% patients in any group) during the extension study were nasopharyngitis, hypertension, and increased blood creatinine (Additional file 1: Table S2). For patients (*n* = 47) whose lesinurad dose decreased from 400 to 200 mg daily per the protocol amendment, there were no notable safety findings comparing post- versus predose change.

In the core study, the exposure-adjusted incidence rates of any TEAE were 90.9 for the 200CONT group and 93.0 for the 400CONT group (Table 2). With longer exposure to lesinurad in the core plus extension, the exposure-adjusted incidence rates for the 200CONT and 400CONT groups were 34.0 and 34.3, respectively. The exposure-adjusted incidence rates of any serious TEAE were 5.1 and 6.6, respectively, in the core study and 6.3 and 6.6 in the core plus extension.

**Table 2:** Summary of exposure-adjusted incidence rates of treatment-emergent adverse events during the core study and the core study + extension study (safety population)

System organ class Preferred term [*n* (exposure-adjusted rate)]	Core study				Core + extension study			
	200CONT (*n* = 64) (PY = 59.4)	200CROSS (*n* = 33) (PY = 30.6)^a^	400CONT (*n* = 65) (PY = 60.2)	400CROSS (*n* = 34) (PY = 31.5)^a^	200CONT (*n* = 64) (PY = 173.6)	200CROSS (*n* = 33) (PY = 61.9)	400CONT (*n* = 65) (PY = 180.6)	400CROSS (*n* = 34) (PY = 61.7)
Any TEAE	54 (90.9)	20 (65.4)	56 (93.0)	29 (92.1)	59 (34.0)	28 (45.2)	62 (34.3)	33 (53.5)
Any serious TEAE	3 (5.1)	1 (3.3)	4 (6.6)	1 (3.2)	11 (6.3)	3 (4.8)	12 (6.6)	5 (8.1)
Any fatal TEAE	0	0	0	0	1 (0.6)	0	0	0
Any renal-related TEAE	4 (6.7)	3 (9.8)	6 (10.0)	0	17 (9.8)	2 (3.2)	17 (9.4)	8 (13.0)
Any serious renal-related TEAE	0	0	0	0	1 (0.6)	0	1 (0.6)	1 (1.6)
Any kidney stone AE	0	0	1 (1.7)	1 (3.2)	3 (1.7)	1 (1.6)	5 (2.8)	2 (3.2)
Any serious kidney stone AE	0	0	0	1 (3.2)	0	0	2 (1.1)	1 (1.6)
sCr elevation 1.5× baseline	3 (5.1)	0	7 (11.6)	2 (6.3)	10 (5.8)	6 (9.7)	18 (10.0)	7 (11.3)
sCr elevation 2.0× baseline	2 (3.4)	0	3 (5.0)	0	6 (3.5)	0	4 (2.2)	4 (6.5)
MACE	0	0	1 (1.7)	0	1 (0.6)	0	2 (1.1)	0
Adjudicated cardiovascular events	3 (5.1)	0	3 (5.0)	0	4 (2.3)	1 (1.6)	4 (2.2)	2 (3.2)

*Abbreviations: CONT* Continuation of lesinurad treatment, *CROSS* Crossover from core study placebo to lesinurad treatment, *MACE* Major adverse cardiovascular event (nonfatal myocardial infraction, nonfatal stroke, cardiovascular death), *PY* Person-years of lesinurad exposure, *sCr* Serum creatinine, *TEAE* Treatment-emergent adverse event

^a^PY are person-years of placebo (febuxostat) exposure

The 200CROSS and 400CROSS groups were not exposed to lesinurad in the core study, so all PYs were calculated as patients with events per 100 PY of placebo (febuxostat) exposure. With exposure to lesinurad in the core plus extension, the exposure-adjusted incidence rates of any TEAE were 45.2 for the 200CROSS group and 53.5 for the 400CROSS group. Exposure-adjusted incidence rates of any serious TEAE were 4.8 and 8.1 for the 200CROSS and 400CROSS groups, respectively.

#### Renal-related adverse events

The proportions of patients who experienced at least one renal-related AE in the extension study were 25.0%, 6.1%, 21.5%, and 23.5% for the 200CONT, 200CROSS, 400CONT, and 400CROSS groups, respectively. The most common individual renal-related AE was increased blood creatinine (15.6%, 3.0%, 13.8%, and 11.8%, respectively). The only renal-related SAEs were experienced by one patient (1.6%) in the 200CONT group (renal impairment), one (1.5%) in the 400CONT group (acute renal failure), and one (2.9%) in the 400CROSS group (acute renal failure). No patients underwent hemodialysis. The exposure-adjusted incidence rates of any renal-related TEAE were 6.7 for the 200CONT group and 10.0 for the 400CONT group in the core study and 9.8 and 9.4, respectively, in the core plus extension (Table 2). The exposure-adjusted incidence rates were 3.2 and 13.0 for the 200CROSS and 400CROSS groups, respectively, in the core plus extension.

Altogether, ten kidney stone TEAEs were reported in ten patients during the extension study, three of which were SAEs (400CONT, 2; 400CROSS, 1). All ten kidney stone TEAEs led to study withdrawal as required by the protocol. The incidence of kidney stone AEs was comparable between the 200CONT plus 200CROSS groups (4.1%; four patients) and the 400CONT plus 400CROSS groups (6.1%; six patients); seven of the ten kidney stone TEAEs occurred in patients randomized to lesinurad in the core study. The exposure-adjusted incidence rates for kidney stones in the core study were 0, 0, 1.7, and 3.2 for the 200CONT, 200CROSS, 400CONT, and 400CROSS groups, respectively, and 1.7, 1.6, 2.8, and 3.2, respectively, in the core plus extension (Table 2).

#### Cardiovascular-related adverse events

During the extension study, events adjudicated by the Cardiovascular Endpoints Adjudication Committee as cardiovascular events occurred in 4.7%, 3.0%, 1.5%, and 5.9% of patients in the 200CONT, 200CROSS, 400CONT, and 400CROSS groups, respectively. There was a MACE in each CONT group and none in the CROSS groups.

The exposure-adjusted incidence rates of patients with events adjudicated as cardiovascular events in the core study were 5.1 for the 200CONT group and 5.0 for the 400CONT group and 2.3 and 2.2, respectively, in the core plus extension (Table 2). The exposure-adjusted incidence rates were 1.6 and 3.2 for the 200CROSS and 400CROSS groups in the core plus extension study. The exposure-adjusted incidence rates for MACE in the core were 0, 0, 1.7, and 0 for the 200CONT, 200CROSS, 400CONT, and 400CROSS groups, respectively, and 0.6, 0, 1.1, and 0, respectively, in the core plus extension and core plus extension.

#### Laboratory evaluations and vital signs

In the extension study, sCr elevation greater than or equal to 1.5 times baseline (baseline defined as the sCr value prior to starting lesinurad) occurred in 15 (15.5%) patients (19 elevations) in the 200CONT and 200CROSS groups and in 21 (21.2%) patients (23 elevations) in the 400CONT and 400CROSS groups (Table 3). Resolution of sCr elevations (defined as sCr less than or equal to 1.2 times baseline) occurred in 57–100% of cases and without an interruption of lesinurad treatment in 57–100% of cases (Table 3).

**Table 3:** Incidence of serum creatinine elevations during the extension study

Serum creatinine criterion	200CONT (*N* = 64) *n* (%)	200CROSS (*N* = 33) *n* (%)	400CONT (*N* = 65) *n* (%)	400CROSS (*N* = 34) *n* (%)	
sCr elevation ≥ 1.5× lesinurad baseline^a^					
Number of patients with elevation	9 (14.1)	6 (18.2)	14 (21.5)	7 (20.6)	
Number of elevations	12	7	15	8	
Number (%) resolutions^b^	10/12 (83.3)	4/7 (57.1)	14/15 (93.3)	8/8 (100)	
Number (%) resolutions after interruption of study medication	3/12 (25.0)	0/7 (0)	5/15 (33.3)	0/8 (0)	
Number (%) resolutions without interruption of study medication	7/12 (58.3)	4/7 (57.1)	9/15 (60.0)	8/8 (100)	
Number (%) unresolved at last visit	2/12 (16.7)	3/7 (42.9)	1/15 (6.7)	0/8 (0)	
sCr elevation ≥ 2.0× lesinurad baseline^a^					
Number of patients with elevation	4 (6.3)	0 (0)	2 (3.1)	4 (11.8)	
Number of elevations	4	0	2	5	
Number (%) resolutions^b^	4/4 (100)	0/0 (0)	2/2 (100)	5/5 (100)	
Number (%) resolutions after interruption of study medication	3/4 (75.3)	0/0 (0)	1/2 (50.0)	0/5 (0)	
Number (%) resolutions without interruption of study medication	1/4 (25.0)	0/0 (0)	1/2 (50.0)	5/5 (100)	
Number (%) unresolved at last visit	0/4 (0)	0/0 (0)	0/2 (0)	0/5 (0)	

*Abbreviations: CONT* Continuation of lesinurad treatment, *CROSS* Crossover from core study placebo to lesinurad treatment, *sCr* serum creatinine

^a^Lesinurad baseline is before starting lesinurad therapy; for CONT groups, at the start of the core study; for CROSS groups, at the start of the extension study

^b^Resolution defined as sCr value ≤ 1.2× baseline following an elevation

sCr elevation greater than or equal to 2.0 times baseline occurred in four (4.1%) patients (four elevations) in the 200CONT plus 200CROSS groups and six (7.1%) patients (seven elevations) in the 400CONT plus 400CROSS groups (Table 3). Resolution of sCr elevations occurred in 100% of cases, 25–100% without an interruption of lesinurad treatment (Table 3).

Exposure-adjusted incidence rates of sCr greater than or equal to 1.5 times baseline were 5.1 and 11.6 for the 200CONT and 400CONT groups, respectively, in the core study and 5.8 and 10.0, respectively, in the core plus extension (Table 2). Exposure-adjusted incidence rates for sCr greater than or equal to 1.5 times baseline in the 200CROSS and 400CROSS groups in the core plus extension were 9.7 and 11.3, respectively. Exposure-adjusted incidence rates of sCr greater than or equal to 2.0 times baseline were 3.4 and 5.0 for the 200CONT and 400CONTgroups, respectively, in the core study and 3.5 and 2.2, respectively, in the core plus extension. Exposure-adjusted incidence rates for sCr greater than or equal to 2.0 times baseline in the 200CROSS and 400CROSS groups in the core plus extension were 0 and 6.5, respectively. Other clinical safety laboratory values and vital signs were generally similar across treatment groups, with no notable changes from baseline in any group.

## Discussion

The goal of gout management is to achieve a sustained lowering in sUA levels, recommended at < 6.0 or < 5.0 mg/dl according to the severity of gout symptoms [1, 2]. Few controlled studies to date have investigated the efficacy of ULTs to maintain sUA levels at target and the effects of sustained sUA lowering on gout symptoms.

In the CRYSTAL phase III trial, after 12 months, combination therapy with lesinurad and febuxostat 80 mg was shown to maintain sUA < 5.0 mg/dl in ~ 57% and ~ 61% of patients in the 200-mg or 400-mg lesinurad dose groups, respectively, compared with ~ 41% of patients treated with febuxostat 80 mg alone [11]. Patients who entered the extension study represent a subset of the core study population: those who were able to tolerate the study medications and who completed the core study treatment. For those in the extension study who continued to receive combination therapy, sUA lowering was sustained without attenuation over 12 months. For those who crossed over from receiving febuxostat alone to receiving combination therapy, the proportions of patients achieving target sUA increased, reaching proportions similar to the groups that continued lesinurad therapy at the end of month 12.

By the end of the core study, there was a numerical increase in the proportion of patients with complete resolution of at least one target tophus with combination therapy compared with febuxostat alone, but differences were not statistically significant [13]. The proportion of patients with complete tophus resolution continued to increase during the extension study, reaching nearly two-thirds of patients by extension month 12. There was also a significant, almost 50% greater, reduction in target tophus area with lesinurad 200 mg plus febuxostat and lesinurad 400 mg plus febuxostat groups compared with febuxostat alone at the end of the core study. Tophus area continued to decrease during the extension study, declining by as much as 78% at extension month 12. The improvement in tophus regression in all groups during the extension was not unexpected, because the mean sUA at the start of the extension was < 6 mg/dl even for the groups receiving febuxostat alone during the core study, and the sUA was maintained or reduced further throughout the extension with the more intensive ULT. However, longer-term treatment may be needed to further dissolve baseline tophi, particularly to demonstrate differences in treatment [15, 16].

Patients who received lesinurad in the core study and continued in the extension study had the lowest adjusted gout flare rate at extension month 12, being lower in the 400CONT group than the 200CONT group. The gout flare rates were higher in patients in the crossover groups where they only had up to 12 months of combination therapy. As expected with the initiation of an effective ULT, rates of gout flares requiring treatment initially increased in patients previously on 12 months of febuxostat monotherapy in the core study when they began lesinurad combination therapy in the extension study. The proportion of patients who had a gout flare requiring treatment generally declined over time in all the treatment groups, demonstrating the benefit of continued sUA lowering.

Gout flares and tophi can seriously impair the quality of life in patients with gout [17–20]. Appropriate ULT that effectively lowers and maintains sUA at target levels can reduce the frequency of gout flares [18, 20, 21] and the size and number of tophi [5, 18, 21], leading to an improved quality of life [22–24]. Although not addressed in the present study, an improvement in quality of life would be consistent with the observed reduction in gout flares and increased tophus resolution as a result of lower sUA levels with lesinurad plus febuxostat treatment. The present results are also consistent with the conclusion that more than 1 year of ULT is required to reduce gout flares and increase tophus resolution, presumably because it takes time to reduce the body urate load [16, 25, 26].

Consistent with safety outcomes in the core study, treatment with lesinurad 200 mg in combination with febuxostat in the core and extension studies was generally well tolerated in patients with tophaceous gout. For those patients who entered the extension study and had tolerated therapy in the core study, some developed renal-related AEs, including sCr elevations during the extension study. Thus, ongoing monitoring of kidney function is important for patients receiving lesinurad therapy on a long-term basis. No new safety signals were observed, because the exposure-adjusted incidence rates of TEAEs in the core plus extension studies were not greater than those in the core study alone. The combination of lesinurad 400 mg plus febuxostat was associated with a higher incidence of sCr elevations than with lesinurad 200 mg plus febuxostat. Resolution of sCr elevations occurred by the last visit in the majority of patients in all treatment groups.

Limitations of the extension study that are shared with the core study include the small number of women in the study and imprecision in the methodology for measuring flares and tophus resolution. Also, patients who entered the extension study are a subset of the core study population: those who were able to tolerate the study medications and who completed the core study treatment. As mentioned above, although there was continued reduction in tophus area with treatment time in this study, treatment for longer than 2 years may be needed to further resolve baseline tophi, particularly to demonstrate differences in treatment [15, 16].

## Conclusions

In this extension study, continued combination therapy with lesinurad and febuxostat maintained lower sUA levels for up to 24 months in patients with tophaceous gout who were unable to achieve target sUA on febuxostat alone. There was a persistent trend toward resolution of tophi and reduction in the rate of flares. No new safety concerns were evident with longer exposure to combination therapy. Renal-related AEs, including sCr elevations, were observed after the first year of treatment, and ongoing monitoring of kidney function is important for patients receiving lesinurad therapy on a long-term basis. These data provide further support for combining a uricosuric with a xanthine oxidase inhibitor in the treatment of patients with tophaceous gout.

## Additional files

Additional file 1: **Table S1.** Renal-related and kidney stone TEAEs. **Table S2.** TEAEs with > 5% of patients in either total treatment group during the extension study (safety population). *CONT* Continuation of lesinurad treatment, *CROSS* Crossover from core study placebo to lesinurad treatment. **Figure S1.** Patient disposition. *Subjects terminated any time during the study. (DOCX 88 kb)

## Abbreviations

AE: Adverse event
CRYSTAL: Combination Treatment Study in Subjects with Tophaceous Gout with Lesinurad and Febuxostat
MACE: Major adverse cardiovascular event
NSAID: Nonsteroidal anti-inflammatory drug
PY: Person-years
SAE: Serious adverse event
sCr: Serum creatinine
sUA: Serum urate
TEAE: Treatment-emergent adverse event
ULT: Urate-lowering therapy
