# Supplementary material for: Efficacy and safety during extended treatment of lesinurad in combination with febuxostat in patients with tophaceous gout: CRYSTAL extension study
Source: Arthritis Res Ther. 2019 Jan 7;21:8. doi: 10.1186/s13075-018-1788-4 (PMC6322285; doi:10.1186/s13075-018-1788-4)
Supplement: Supplementary file 1 — Table S1. Renal-related and kidney stone TEAEs. Table S2. TEAEs with > 5% of patients in either total treatment group during the extension study (safety population). CONT Continuation of lesinurad treatment, CROSS Crossover from core study placebo to lesinurad treatment. Figure S1. Patient disposition. *Subjects terminated any time during the study. (DOCX 88 kb) [file 13075_2018_1788_MOESM1_ESM.docx]

**Table S1** Renal-related and kidney stone TEAEs

| **Renal-related TEAEs** | **Kidney stone TEAEs** |
| --- | --- |
| - Acute prerenal failure - Anuria - Azotemia - Blood creatinine abnormal - Blood creatinine increased - Blood urea abnormal - Blood urea increased - Blood urea nitrogen/creatinine ratio increased - Creatinine renal clearance abnormal - Creatinine renal clearance decreased - Cystatin C abnormal - Cystatin C increased - Glomerular filtration rate abnormal - Glomerular filtration rate decreased - Hypercreatininemia - Inulin renal clearance abnormal - Inulin renal clearance decreased - Nephropathy - Nephropathy toxic - Obstructive uropathy - Oliguria - Postrenal failure - Renal cortical necrosis - Renal failure - Renal failure acute - Renal failure chronic - Renal function test abnormal - Renal impairment - Renal injury - Renal papillary necrosis - Renal tubular atrophy - Renal tubular disorder - Renal tubular necrosis - Urate nephropathy - Urea renal clearance decreased - Urine output decreased | - Calculus bladder - Calculus ureteric - Calculus urethral - Calculus urinary - Nephrolithiasis - Renal stone removal - Stag horn calculus - Ureteric calculus removal - Ureterolithotomy - Urinary calculus removal - Urinary stone analysis |

**Fig. S1** Patient disposition


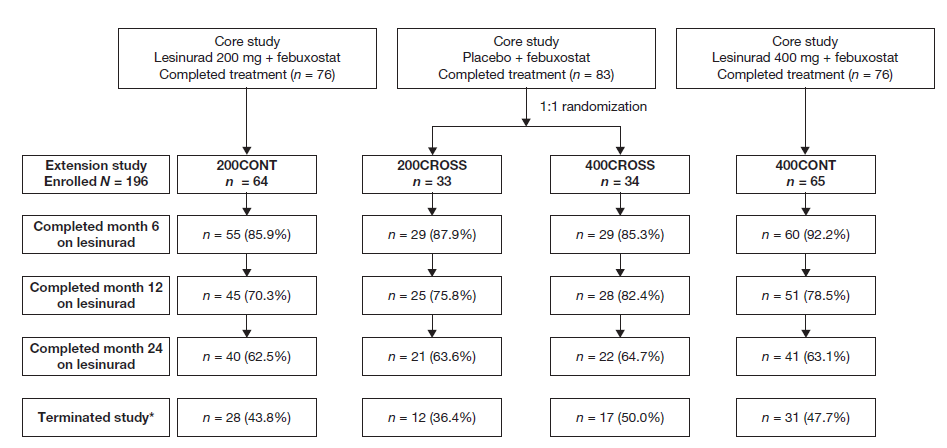


*Subjects terminated any time during the study.

**Table S2** Treatment-emergent adverse events with > 5% of patients in either total treatment group during the extension study (safety population)

| System organ class  Preferred term | 200CONT (*n* = 64) *n* (%) | 200CROSS (*n* = 33) *n* (%) | 400CONT (*n* = 65) *n* (%) | 400CROSS (*n* = 34) *n* (%) |
| --- | --- | --- | --- | --- |
| Nasopharyngitis | 4 (6.3) | 2 (6.1) | 6 (9.2) | 8 (23.5) |
| Hypertension | 4 (6.3) | 2 (6.1) | 5 (7.7) | 6 (17.6) |
| Blood creatinine increased | 10 (15.6) | 1 (3.0) | 9 (13.8) | 4 (11.8) |
| Sinusitis | 3 (4.7) | 5 (15.2) | 3 (4.6) | 2 (5.9) |
| Headache | 2 (3.1) | 3 (9.1) | 2 (3.1) | 5 (14.7) |
| Bronchitis | 8 (12.5) | 3 (9.1) | 3 (4.6) | 1 (2.9) |
| Arthralgia | 3 (4.7) | 1 (3.0) | 2 (3.1) | 4 (11.8) |
| Upper respiratory tract infection | 6 (9.4) | 2 (6.1) | 4 (6.2) | 2 (5.9) |
| Urinary tract infection | 6 (9.4) | 0 | 4 (6.2) | 1 (2.9) |
| Osteoarthritis | 2 (3.1) | 3 (9.1) | 1 (1.5) | 3 (8.8) |
| Muscle strain | 3 (4.7) | 3 (9.1) | 0 | 0 |
| Benign prostatic hyperplasia | 0 | 1 (3.0) | 3 (4.6) | 3 (8.8) |
| Laceration | 0 | 0 | 3 (4.6) | 3 (8.8) |
| Diabetes mellitus | 0 | 0 | 2 (3.1) | 3 (8.8) |
| Creatinine renal clearance decreased | 4 (6.3) | 1 (3.0) | 2 (3.1) | 1 (2.9) |
| Gastroesophageal reflux disease | 4 (6.3) | 1 (3.0) | 1 (1.5) | 0 |
| Back pain | 4 (6.3) | 2 (6.1) | 3 (4.6) | 2 (5.9) |
| Influenza | 0 | 0 | 4 (6.2) | 2 (5.9) |
| Depression | 0 | 0 | 4 (6.2) | 1 (2.9) |
| Toothache | 3 (4.7) | 1 (3.0) | 4 (6.2) | 1 (2.9) |
| Musculoskeletal pain | 3 (4.7) | 2 (6.1) | 1 (1.5) | 1 (2.9) |
| Nephrolithiasis | 3 (4.7) | 1 (3.0) | 3 (4.6) | 2 (5.9) |

*CONT* continuation of lesinurad treatment, *CROSS* crossover from core study placebo to lesinurad treatment
